# Written Language Acquisition Is Both Shaped by and Has an Impact on Brain Functioning and Cognition

**DOI:** 10.3389/fnhum.2022.819956

**Published:** 2022-06-10

**Authors:** Felipe Pegado

**Affiliations:** Aix-Marseille University, CNRS, LPC, Marseille, France

**Keywords:** learning, brain, cognition, plasticity, reading, writing

## Abstract

Spoken language is a distinctive trace of our species and it is naturally acquired during infancy. Written language, in contrast, is artificial, and the correspondences between arbitrary visual symbols and the spoken language for reading and writing should be explicitly learned with external help. In this paper, I present several examples of how written language acquisition is both shaped by and has an impact on brain function and cognition. They show in one hand how our phylogenetic legacy influences education and on the other hand how ontogenetic needs for education can rapidly subdue deeply rooted neurocognitive mechanisms. The understanding of this bidirectional influences provides a more dynamic view of how plasticity interfaces phylogeny and ontogeny in human learning, with implications for both neurosciences and education.

## The Natural Acquisition of Spoken Language in Humans

Elementary forms of communication, by using vocalizations or actions conveying communicative messages, are observed in non-human primates (Meguerditchian and Vauclair, 2014). Spoken language, however, is unique to humans. Spoken language enables sophisticated communication of ideas, including abstract concepts. Language can be viewed as intrinsically related to our thoughts, influencing our view of the world, our cognition (i.e., “the mental processes of acquiring knowledge such as perception, reasoning, memory, etc.”): the so-called the “Sapir-Whorf” hypothesis, also known as the “linguistic relativity hypothesis” (Kay and Kempton, 1984; Siok et al., 2009). Radical versions of the Sapir-Whorf hypothesis, i.e., language *fully determines* our cognition is probably less considered nowadays (Kay and Kempton, 1984) than its moderate version, i.e., language can *modulate/partially influence* our cognition, in a variety of domains, from color perception (Siok et al., 2009) and motor learning (Foerster et al., 2020) to motion, number, time, and object processing (for a review, see Pae, 2020). Further, the degree of language influence on cognition could be directly related to the degree of uncertainty of the inferences performed (Regier and Xu, 2017).

In fact, Darwin had already pointed our “natural instinct” to acquire language, just requiring sufficient time, exposure and interaction to naturally develop it (Pinker, 2003). This instinct is refereed by Chomsky as our universal “language acquisition device,” being present across all human cultures in the world. One fundamental characteristic of human language highlighted by Saussure is the association of arbitrary vocal sounds to specific meanings, i.e., the *arbitrariness* between a signifier and a signified (Holdcroft, 1991). Another characteristic is its *recursiveness*, i.e., the capacity to produce infinite combinations from finite units (Hauser et al., 2002). This, at different hierarchical levels: phonemes forming words and words composing sentences (Martinet, 1957).

Despite the complexity of grammatical structure and syntax, humans acquire language during early development (infancy) without explicit teaching. As a matter of fact, from the beginning of the third trimester of gestation, human fetus can already hear, starting to be exposed to spoken language from inside the uterus, especially at lower frequencies (Gerhardt and Abrams, 1996). After birth, newborn babies started to be exposed to the whole spectrum of language frequencies and by the age of 12 months-old, they can distinguish between different phonemes-in-syllables present in their mother language. However, they also lose at this point the ability to discriminate new phonemes that are not present in their mother language (e.g., lack of distinction between “ra” and “la” in Japanese). This can be observed both at the behavioral (Kuhl et al., 2006) and brain response levels (Kuhl, 2010). Further, studies show a *sensitive* period (defined here as a “window of opportunity,” instead of a binary possible vs. impossible learning period for which the term “critical period” will be reserved) for the mastery of a second language, particularly in terms of foreign accent (Flege et al., 1995) and syntax (Johnson and Newport, 1989; Weber-Fox and Neville, 1996), up to the adolescence, although individual differences can be detected across subjects and results can depend on task exigences (Flege et al., 1995). In addition, the ideal phase to learn a second language seems to be before the age of 10 years-old but at least no later than 17 years-old (Hartshorne et al., 2018).

Despite the natural instinct for learning to speak in humans, speech production requires a very complex coordination of muscular activity to produce the precise fast changing sequences of linguistic sounds. Thus, it is not surprising that difficulties in this complex machinery can emerge, such as different forms of stuttering (Prasse and Kikano, 2008). Stuttering represents an important source of social distress and its incidence has been estimated to 5% of the general population or even higher (Yairi and Ambrose, 2013). Importantly, however, the plasticity induced by further learning (with but even without formal therapy) reduces the persistence of stuttering over the life span to below 1%, which can be partially modulated by genetic factors inducing “persistent” vs. “recovered” outcomes (Yairi and Ambrose, 2013).

The neural basis of speech includes a lateralization of language processing to the left hemisphere in the vast majority of persons (even among left handers), engaging in particular the perisylvian regions, including the primary and secondary auditory cortex. The so-called “Broca’s area” in the inferior frontal cortex plays an important role in speech production but also in speech understanding, particularly under difficult conditions such as in noisy environments, i.e., the “cocktail party” paradigm (Wu et al., 2014). Further, it has also been suggested that Broca’s area is directly linked to syntax processing (Friederici, 2018).

## The Artificial Acquisition of Written Language in Humans

In a clear contrast with the natural acquisition of spoken language, the acquisition of written language is artificial. Children do not learn to read and write by themselves. They need explicit instructions from educators (and/or parents). Furthermore, relative to the early acquisition of spoken language, written language is acquired much later in the development, typically around 6 or 7 years of age. Finally, relative to speech ability, writing systems emerged much later in our evolutionary history (in a few thousands of years ago instead of the putative hundreds of thousands of years). Thus, these three key characteristics of written language learning (artificial, acquired later in ontogeny, and in phylogeny) distinguish it clearly from spoken language learning.

Indeed, the first writing systems emerged about 5,000 years ago. Among them were the Sumerian cuneiform, the Chinese characters and the Hieroglyph scripts. Today a large variety of scripts are present in the world, including *alphabetic* systems, such as the Latin alphabet, which allows a fine-grained translation of the spoken language (phonemes) into arbitrary visual symbols (graphemes), and is now largely used around the world. The grapheme-phoneme mapping empowers literates to “listen with the eyes,” as you are doing right now.

Writing can be considered one of the greatest human inventions, enabling to “print our language,” our thoughts, our memories, in a physical support. Written language allows the transfer of knowledge across generations and social groups, over time and space. The emergence of writing delimitates the end of the long *prehistorical* period of humankind- when only oral transmission of knowledge was possible- and the beginning of human *history*.

## Written Language Acquisition Is Shaped by Brain Functioning and Cognition

Learning to read an alphabetic script requires the understanding of the correspondence between letter combinations and the sounds of language (i.e., the alphabetic principle). This linguistic audio-visual mapping learning is constrained by the brain functioning, defined here as the physiological neural mechanisms selected by evolution. For instance, as other primates, the human visual system presents a hierarchical organization, with low-level visual areas processing low-level features of images and high-level areas representing “visual objects” in a more abstract way. In fact, high-level areas specializes for different types of images such as places, faces and objects, in a mesial-to-lateral patches organization (Ishai et al., 2000; Hasson et al., 2003; Dehaene-Lambertz et al., 2018). Furthermore, the representation of objects in high-level visual cortex is view invariant (Wallis and Rolls, 1997; Freiwald and Tsao, 2010). For instance, tolerance for changes in perspectives (rotations of objects) or changes in size (an object getting closer or more distant) are naturally useful for object recognition in different situations and accordingly equivalent brain responses are found for different degrees of changes in high-level visual cortex of primates and humans (Tanaka, 1997; Li and DiCarlo, 2008). The neural mechanisms enabling invariant representations of visual objects (size and shape invariance) in the visual cortex have a direct impact on the visual processing of letters.

As a matter of fact, to read, children must recognize a new class of visual objects: letters. Literacy induces a specialization of the ventral visual cortex in the temporal lobe of the left hemisphere to process orthographic stimuli, a region commonly referred as the Visual Word Form Area (VWFA) (Cohen et al., 2000). The VWFA is typically located between the faces and object’s patches and is supposed to emerge from a “competition” for cortex territory: learning to recognize letters induces a preference for letters in a small part of the left ventral temporal cortex, instead of a preference for faces (Dehaene et al., 2010) or objects (Dehaene-Lambertz et al., 2018). Therefore, the VWFA receives the heritage of the common functional properties of these high-level visual areas, e.g., skilled readers show a *size-invariant* and a *shape invariant* representation of letters (Quiroga et al., 2005; Chauncey et al., 2008; Han et al., 2020). It has been proposed the *neuronal recycling hypothesis* to explain the plastic changes related to cultural inventions such as reading and arithmetic, that “invade” cortical circuits shaped by evolution for other uses (Dehaene and Cohen, 2007). However, it is unclear if these plastic changes present any special characteristic or if they obey the same general principles of neuronal plasticity as in other situations such as early blindness for instance, where the visual cortex plastically adapts to process other sensory stimuli (Renier et al., 2014), deviating from their original function.

Another general property of our primate visual system is its capacity to process information in *parallel* (Nakayama and Silverman, 1986; Cave and Wolfe, 1990; Nassi and Callaway, 2009). This property has also direct consequences in reading: written language processing, contrary to the serial processing of spoken language, can also operate in a more parallel way. Skilled readers show *parallel* processing of letters-in-words, both processing letters embedded in multi-letter graphemes (e.g., ch or ph) or syllables as single units (Rastle and Coltheart, 1998; Rey et al., 1998, 2000; Commissaire et al., 2018). Furthermore, skilled readers show a lack of “word length effect” (i.e., short words are read in the same speed as long words) in visual word processing, which suggests that all letters in a word are processed in parallel (Ziegler et al., 2001, 2003). Furthermore, reading performance of skilled readers is well preserved even when the order of letters is slightly modified (e.g., *pitcure* as opposed to *picture;* or within a sentence context: “it deosn’t mttaer in waht oredr the ltteers in a wrod are”). This again suggest that skilled readers abandon strict serial processing in favor of more parallel orthographic processing (Grainger and Ziegler, 2011; Grainger et al., 2016). Interestingly, recently developed behavioral paradigms were able to challenge the traditional view that skilled readers are limited to a serial *word-by-word* reading. New studies provide evidence for *parallel word* processing. For instance, in the sentence “Do like you this idea?” did you noticed that the second and third words were transposed? Skilled readers demonstrate more difficulty to detect transposed words in a line of text, relative to a control condition such as replacing two words (Mirault et al., 2018). This “transposed-word” effect is modulated by *lexical* status, with stronger effects for words than pseudowords (Pegado and Grainger, 2019). Further, it is also modulated by *syntactic* structure, with stronger effects for grammatically correct sequences of words relative to scramble versions of these same words (Pegado and Grainger, 2020a,b). Finally, brain imaging also show that two words can be processed in parallel in subparts of the VWFA of skilled readers (White et al., 2019).

Taken together, these results suggest that deeply rooted visual mechanisms already present in our primate lineage such as shape invariance, size invariance and parallel processing for images in general, modulates the visual processing of written language. Thus, the functioning of our visual system determines how we can process letters and words visually. Note the different for instance in the auditory system that process sounds of language more serially given the intrinsic characteristics in this modality (serial time-course of speech).

## Written Language Acquisition Also Shapes Brain Functioning and Cognition

Importantly, not only the physiology of brain functioning impacts written language acquisition. The acquisition of written language is also able to modulate brain functioning and cognition.

Interestingly, an extension of the linguistic relativity hypothesis, from spoken language to written language, has been recently evoked as the *script relativity hypothesis*, stating that “Just like linguistic relativity that postulates that habitual language use results in a unique set of habitual thought and thinking patterns, habitual reading of a particular script has the great potential to yield unique thought processes or patterns in the reader’s mind as an embodied experience” (Pae, 2020). She proposes that differences in script systems could explain cognitive differences among three East-Asian nations (Japan, Korea, China) in one hand and between the East and the West on the other hand, in a variety of domains such as attention, perception, problem-solving strategies, and rhetorical structures. For instance, Westerners’ visual span is greater toward the right side, while readers of Hebrew (i.e., right-to-left readers), show the inverted asymmetry (i.e., to the left side) (Pollatsek et al., 1981). Further, Chinese readers, whose characters are much denser than those in English (Latin alphabet), present shorter saccades and a reduced visual span (Pae, 2020). These results highlight the importance of considering potential differences (and universal aspects) of the neural basis of reading and its impact on cognition across scripts (Nakamura et al., 2012).

In fact, the building of the linguistic audio-visual mapping for written language produces deep consequences at the cognitive and brain levels (van Atteveldt et al., 2004). Fluent readers activate their auditory cortex from *visual* inputs of words, reaching equivalent activation levels as those produced by *auditory* inputs (Dehaene et al., 2010), confirming that literates can literally “listen with the eyes.” Literacy also enables the activation of the VWFA from *auditory* stimuli in a lexical decision task (i.e., judging if a stimulus is a word or a non-word) (Dehaene et al., 2010). Note that, in this type of task, orthographic information is useful to disentangle words from pseudowords and in fact the VWFA activation seems to reflect their recruitment. However, there is no activation in the VWFA during *passive* listening of sentences, from the same participants. Thus, the acquisition of the artificial audio-visual linguistic mapping (literacy acquisition) enables a bidirectional activation between the visual and auditory systems, with an asymmetric effect: while the auditory activation from visual inputs of words was automatic and intense in fluent readers, the visual activation from auditory linguistic inputs did not occur automatically but only when orthographic information was task relevant.

Furthermore, visual processing of letters is rapidly (<200 ms) directed to the left hemisphere, which is the “language hemisphere” for most people, as indexed by an electro- physiological marker (N170), suggesting a highly automatized process (Pegado et al., 2014a).

Importantly, in addition to the previously described bidirectional influences between the visual and auditory systems related to literacy acquisition, literacy also induces changes in *auditory* representations themselves. Illiterates are able to segment and manipulate syllables of spoken language. However literates, but not illiterates, are able to detect and mentally manipulate the smallest units of spoken language, i.e., the phonemes (e.g., in a task of deleting phonemes in words), an ability referred as “phonemic awareness” (Morais et al., 1979, 1987). This is a good example of how learning the linguistic audio-visual mapping can concretely change cognition (linguistic and script relativity hypotheses). Accordingly, literacy induces stronger activations in a phonological area (*Planum Temporale*), perhaps related to the refinement of phonological representations induced by the grapheme-phoneme mappings (Dehaene et al., 2010). In fact, the results of Morais can be viewed in more general terms as following: learning in one sensory modality (auditory) can be enhanced by mappings with other sensory modalities (visual), relative to unisensory learning alone, as for illiterates that only possess spoken language. This same “multisensory advantage” is also found in more generic audio-visual paradigms outside the language domain (Shams and Seitz, 2008). Furthermore, the linguistic audio-visual mapping is not the only mapping induced by literacy, as children typically also learn to *write* letters and words, creating a connection with the motor writing gestures system. As a result, automatic activations of the premotor cortex, anterior to the hand-related motor cortex (known as the “Exner area”) are observed when skilled readers visually perceive letters being written during neuroimaging experiments (Longcamp et al., 2003; Nakamura et al., 2012). Interestingly, arbitrary audio-visual mappings, as those created for reading, can be *reinforced* by adding a third modality of letter’s representations (e.g., haptic or grapho-motor) (Fredembach et al., 2009; Bara and Gentaz, 2011; Labat et al., 2015).

Another example of the driving force of literacy training plastically modulating neural mechanisms is provided by the case of *mirror invariance*. Mirror invariance is a physiological visual mechanism that enables the recognition of mirror images. It emerges early in human development (3–4 months old babies) (Bornstein et al., 1978) and is also shared with non-human primates, such as monkeys (Noble, 1966; Logothetis and Pauls, 1995; Rollenhagen and Olson, 2000), suggesting that it is a phylogenetically old mechanism of at least ∼25 million years old, given the common ancestor between us and old world monkeys (Stevens et al., 2013). Perhaps even older, given that this mechanism is also present in phylogenetically more distant animals such as pigeons (Mello, 1965) and cephalopods (Sutherland, 1957). In any case, while mirror invariance is useful in the natural world to recognize images from left and right perspectives, it can create “mirror confusion” for letters, as letters have a fixed left/right orientation and some are even mirror-images of others in the Latin alphabet such as p-q or d-b and to a certain extend s-z. This mirror confusion is a pervasive difficulty in the beginning of literacy acquisition, and can persist even after 2–3 years of literacy practice (Cornell, 1985; McIntosh et al., 2018). However, reading training can progressively mitigate the impact of mirror invariance, with skilled readers exhibiting fast discrimination between left/right orientation of letters, at the perceptual level (Pegado et al., 2014b) and at the brain responses level (Pegado et al., 2011, 2014a). Interestingly, the VWFA exhibits mirror invariance for images in general but mirror discrimination for letters in skilled readers, suggesting a selective modulation for orthographic stimuli (Pegado et al., 2011). This effect of literacy takes place at early stages of visual processing time course (<150 ms), confirming the automaticity aspect of this learning (Pegado et al., 2014a).

**FIGURE 1:**
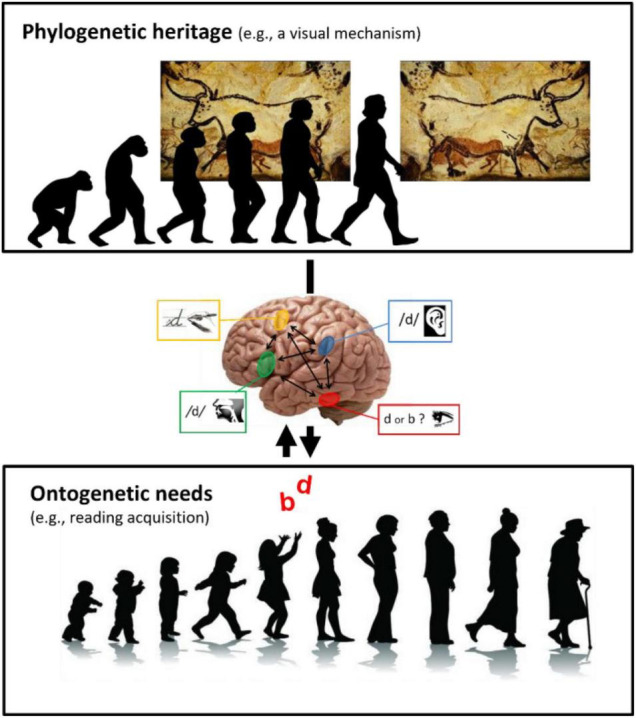
The plasticity of brain functioning and cognition at the interface between phylogenetic heritage and ontogenetic needs in education. The heritage of cognitive and neural mechanisms from evolution has an impact on education (physiological constraints for learning). On the other hand, educational needs can also plastically subdue ancient mechanisms (double arrow). **(Upper)** Illustration showing that *Homo sapiens* (at right) inherited from evolution neural mechanisms such as “mirror invariance,” which enables the recognition of mirror-inverted versions of images as this painting of Lascaux’s cave. **(Middle)** A simplified version of a conceptual model hypothesizing how “mirror invariance” can be inhibited during literacy acquisition (Pegado et al., 2014c). **(Lower)** Despite its usefulness in the natural world, “mirror invariance” can hinders reading fluency acquisition by creating confusion between mirror-letters such as “b” and “d” and should thus be inhibited for fluent reading. Research shows that this ontogenetic need in education can drive plastic changes in human brain and cognition, subduing phylogenetic legacy.

This example clearly shows how neurocognitive plasticity in humans operates at the interface between phylogenetic heritage and ontogenetic needs for education (Figure 1).

One question can then be asked: If the visual recognition system naturally “ignores” letters’ orientation, how literacy inhibits mirror invariance for letters? One hypothesis is that mirror-letter pairs (b/d or p/q), can be disentangled by top-down information from other sensory (auditory) and motor systems (writing and speech production) (Pegado et al., 2014c). Note that no systematic training to differentiate mirror-letters is typically promoted at school. Thus, it is possible that mirror discrimination is only learned incidentally and slowly, as suggested by the long persistence (up to 2–3 years) of mirror confusion for letters (Cornell, 1985; McIntosh et al., 2018).

Interestingly, studies in congenital blind and sight braille readers demonstrate mirror discrimination learning in the tactile modality (de Heering et al., 2018; de Heering and Kolinsky, 2019). Further, automatic mirror discrimination is also observed for the *visual* presentation of braille letters in sigh readers. These results suggest that the tactile representation of letters could have helped the visual system to discriminate braille letter’s orientation. Finally, investigations in dyslexics show persistent mirror invariance for letters (Orton, 1937; Lachmann and Geyer, 2003; Fernandes and Leite, 2017). This persistency is likely to be a consequence of deficient mappings for reading. Taken together, these results suggest that several systems could assist the visual system in the learning process of mirror discrimination for letters.

## Can Education Be Optimized by Taking Advantage of Brain Functioning and Cognition?

**FIGURE 2:**
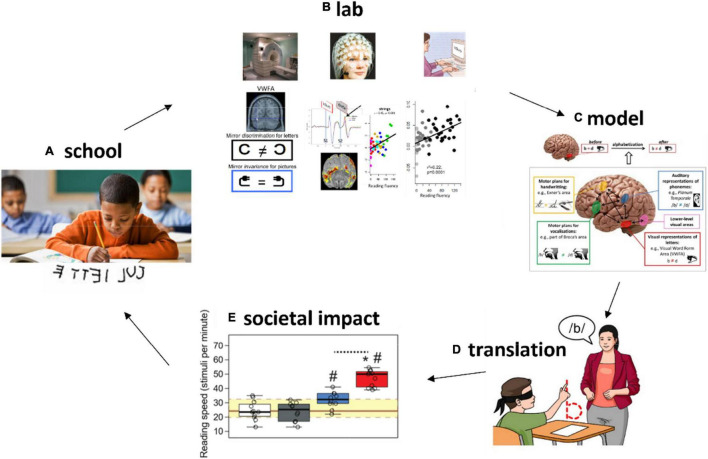
Understanding plastic changes related to education in the lab to improve learning at school. **(A)** Learning difficulties (e.g., mirror confusion for letters) can be understood with sensitive and well-controlled methods in cognitive and neuroscience experiments in the laboratory **(B)**. Conceptual or computational models explaining the learning process **(C)** can be proposed and refined. This understanding opens the door for **(D)** translations into innovative teaching methods that can be tested with rigorous methods such as randomized controlled trials with placebo-like control groups. This brings the potential for real-life improvements in learning (societal impact) **(E)**.

Despite the suggestive results about the importance of other systems to inhibit mirror invariance in the visual system, this hypothesis needed to be confirmed in a more direct way. A recent work addressed this point by using a causal approach (Torres et al., 2021). Placebo-controlled randomized controlled trials (RCTs) were applied in first graders, at school, to probe the impact of a targeted training to distinguish mirror-letters by maximizing mappings across systems, for each of these letters. To potentiate the learning effects, an interaction with memory systems was manipulated in the form of post-training naps (Lemos et al., 2014; Cabral et al., 2018), as sleep is involved in the physiology of memory consolidation. The intervention had a deep impact reducing mirror confusion, improving the visual perception of letters and writing. Furthermore, the combined intervention had a tremendous impact on reading fluency, increasing reading speed by a factor of two, showing that this ancient visual mechanism represents an important “leash” for reading fluency acquisition. On the other hand, it demonstrates the speed of cognitive plasticity in humans, able to inhibit a ∼ 25 million years-old mechanism (or more) in just 3 weeks of training (30 min/day). This is a good example of how the understanding of human physiology can help in the optimization of teaching methods, better fitting the biological constraints received from evolution, and exploiting our extreme plasticity to modulate brain function and cognition (Figure 2).

## Data Availability Statement

The original contributions presented in the study are included in the article/supplementary material, further inquiries can be directed to the corresponding author/s.

## Author Contributions

FP conceived and wrote the manuscript.

## Conflict of Interest

The author declares that the research was conducted in the absence of any commercial or financial relationships that could be construed as a potential conflict of interest.

## Publisher’s Note

All claims expressed in this article are solely those of the authors and do not necessarily represent those of their affiliated organizations, or those of the publisher, the editors and the reviewers. Any product that may be evaluated in this article, or claim that may be made by its manufacturer, is not guaranteed or endorsed by the publisher.
